# Intracranial pressure- and cerebral perfusion pressure threshold-insults in relation to cerebral energy metabolism in aneurysmal subarachnoid hemorrhage

**DOI:** 10.1007/s00701-022-05169-y

**Published:** 2022-03-01

**Authors:** Teodor Svedung Wettervik, Anders Hånell, Timothy Howells, Elisabeth Ronne-Engström, Anders Lewén, Per Enblad

**Affiliations:** Department of Neuroscience, Section of Neurosurgery, Uppsala University, 751 85 Uppsala, Sweden

**Keywords:** Aneurysmal subarachnoid hemorrhage, Cerebral perfusion pressure, Intracranial pressure, Microdialysis, Neurointensive care

## Abstract

**Background:**

The aim was to investigate the association between intracranial pressure (ICP)- and cerebral perfusion pressure (CPP) threshold-insults in relation to cerebral energy metabolism and clinical outcome after aneurysmal subarachnoid hemorrhage (aSAH).

**Methods:**

In this retrospective study, 75 aSAH patients treated in the neurointensive care unit, Uppsala, Sweden, 2008–2018, with ICP and cerebral microdialysis (MD) monitoring were included. The first 10 days were divided into early (day 1–3), early vasospasm (day 4–6.5), and late vasospasm phase (day 6.5–10). The monitoring time (%) of ICP insults (> 20 mmHg and > 25 mmHg), CPP insults (< 60 mmHg, < 70 mmHg, < 80 mmHg, and < 90 mmHg), and autoregulatory CPP optimum (CPPopt) insults (∆CPPopt = CPP-CPPopt <  − 10 mmHg, ∆CPPopt > 10 mmHg, and within the optimal interval ∆CPPopt ± 10 mmHg) were calculated in each phase.

**Results:**

Higher percent of ICP above the 20 mmHg and 25 mmHg thresholds correlated with lower MD-glucose and increased MD-lactate-pyruvate ratio (LPR), particularly in the vasospasm phases. Higher percentage of CPP below all four thresholds (60/70/80//90 mmHg) also correlated with a MD pattern of poor cerebral substrate supply (MD-LPR > 40 and MD-pyruvate < 120 µM) in the vasospasm phase and higher burden of CPP below 60 mmHg was independently associated with higher MD-LPR in the late vasospasm phase. Higher percentage of CPP deviation from CPPopt did not correlate with worse cerebral energy metabolism. Higher burden of CPP-insults below all fixed thresholds in both vasospasm phases were associated with worse clinical outcome. The percentage of ICP-insults and CPP close to CPPopt were not associated with clinical outcome.

**Conclusions:**

Keeping ICP below 20 mmHg and CPP at least above 60 mmHg may improve cerebral energy metabolism and clinical outcome.

**Supplementary Information:**

The online version contains supplementary material available at 10.1007/s00701-022-05169-y.

## Introduction

Intracranial pressure (ICP) and cerebral perfusions pressure (CPP) are the two main treatment variables in the neurointensive care (NIC) of patients with severe aneurysmal subarachnoid hemorrhage (aSAH). For aSAH, the NIC targets for these variables are in clinical practice often extrapolated from traumatic brain injury (TBI) protocols. The current guidelines for TBI suggest keeping ICP below 22 mmHg and maintaining CPP between 60 and 70 mmHg [3]. aSAH patients commonly exhibit intracranial hypertension and cerebral ischemia [26], but there are currently no established aSAH guideline recommendations for ICP and CPP [5, 8]. There is some support for extrapolating the targeted thresholds from TBI to aSAH, as ICP above 20 mmHg seems similarly dangerous and associated with unfavorable clinical outcome in both brain injury conditions [10, 18, 22, 26, 32]. However, whereas a CPP-target within 60–70 mmHg is associated with favorable outcome in TBI [3, 27], CPP above 70 mmHg and higher is associated with a reduced risk of clinical deterioration and higher chance of favorable outcome in aSAH [22, 26]. In addition to potential disease-specific-fixed target thresholds, findings in TBI indicate that continuous, dynamic, patient-specific CPP-targets based on the concurrently optimal autoregulatory status (CPPopt) may be beneficial [1, 24, 26, 27, 30]. Keeping CPP close to CPPopt is associated with better brain tissue oxygenation [14], cerebral energy metabolism [30], and clinical outcome [1, 24, 27, 30] in TBI. In aSAH, recent studies demonstrated that CPP close to CPPopt was associated with a reduced rate of cerebral ischemia [15], but not with better clinical outcome [26].

Altogether, aSAH is a different disease entity as compared with TBI. aSAH patients often exhibit pre-existing cerebrovascular disese predisposing for the cerebral aneurysm that has ruptured and the acute phase is particularly characterized by development of hydrocephalus and cerebral vasospasm. To better understand the effects of ICP- and CPP-insults on the cerebral environment in aSAH patients, there is a need for high-resolution multimodality studies. The primary aim of this study was to determine the association between ICP- and CPP-threshold insults and cerebral energy metabolism, as assessed with the cerebral microdialysis, in aSAH. The secondary aim was to determine the association of these insults with clinical outcome.

## Methods

### Patients

Patients with aSAH admitted to the Department of Neurosurgery at the University Hospital in Uppsala, Sweden, 2008–2018, were eligible for this study. Out of 605 adult patients with SAH and intracranial pressure (ICP) monitoring, 194 SAH patients were monitored with cerebral microdialysis (MD). The first 10 days after ictus were divided into three phases (early phase, early vasospasm phase, and late vasospasm phase) for temporal analyses (see “Data acquisition and analyses” section) and those 75 patients who had ICP and MD monitoring data in all three phases and who did not develop total brain infarction the first 10 days were included in the study (Supplementary Fig. 1). This patient cohort was hence based on the subgroup of patients with MD monitoring from a larger patient population that was studied in a previous study on ICP-/CPP-thresholds in relation to outcome [26].

### Treatment protocol

Patients were managed in accordance with our standardized treatment protocol, which has been described in detail in previous studies [22, 25, 26]. Briefly, our treatment goals were ICP ≤ 20 mm Hg, CPP ≥ 60 mm Hg, systolic blood pressure > 100 mm Hg, pO_2_ > 12 kPa, arterial glucose 5–10 mmol/L (mM), electrolytes within normal ranges, slight hypervolemia with 0 fluid balance after aneurysm occlusion, and body temperature < 38 °C.

Unconscious (Glasgow Coma Scale Motor score (GCS M) < 6) patients were intubated and mechanically ventilated. Propofol was given for sedation and morphine for analgesia. Aneurysms were early occluded, by endovascular embolization or surgical clipping. An external ventricular drain (EVD) was inserted to monitor ICP in unconscious (GCS M < 6) patients. If ICP was above 20 mmHg, the EVD was opened at a drainage level of 15 mmHg. Higher ICP-thresholds such as 20 mmHg for EVD-opening were sometimes used for patients with brain edema or intrventricular clots due to the risk of slit ventricles which may cause false ICP readings. Open EVDs were occasionally closed even in these patients during periods of nursing or wake-up tests. Thiopental infusion and/or decompressive craniectomy (DC) were last-tier treatments for refractory intracranial hypertension. Arterial blood pressure (ABP) was chiefly maintained with fluids and vasopressors were only used if ABP/CPP still remained below the target thresholds. Dobutamine was used as the first line therapy for inotropic support and norepinephrine was used as a second line therapy for further vasopressor support. Nimodipine was given to all patients after NIC admission. Delayed ischemic neurological deficits (DIND) were defined as a new-onset of focal neurological deficit or deterioration in consciousness, not explained by, e.g., hydrocephalus, re-bleeding, or meningitis. If there was no manifest cerebral infarction on computed tomography (CT), a HHH (hypertension, hypervolemia, and hemodilution)-therapy was initiated. HHH-therapy included supine position, colloid fluids to increase the intravascular volume using albumin and dextran solutions, and moderately elevated systolic blood pressure target above 140 mmHg [9].

### Outcome

Clinical outcome was evaluated according to the Extended Glasgow Outcome Scale (GOS-E) 12 months after ictus [28, 31], by trained personnel using structured telephone interviews. GOS-E has eight categories of outcome, from death (1) to upper good recovery (8). Clinical outcome was dichotomized as favorable/unfavorable (GOS-E 5–8/1–4).

### Data acquisition and analyses

ICP was monitored with an EVD system (HanniSet, Xtrans, Smith Medical GmbH, Glasbrunn, Germany). ABP was monitored invasively in the radial artery at heart level. A distinction between open and closed EVD was not done in the data analysis, since ICP was continuously measured also when the system was open and the drainage level adjusted according to the measured values to obtain the prescribed ICP. Physiological data were collected at 100 Hz using the Odin software [11]. Pressure reactivity index (PRx) was calculated as the 5 min correlation of 10 s averages of ICP and MAP [6, 27]. CPPopt was calculated continuously, minute-by-minute, as the CPP in a U-shaped curve with the concurrently lowest PRx the last 4 h [1]. CPPopt values were available during 54% of the monitoring time the first 10 days for all patients and daily CPPopt values could be calculated for 90–96% of the patients, depending on the day.

Cerebral energy metabolism was monitored with the 70 High Cut-Off MD catheter with a membrane length of 10 mm and a membrane cut-off of 20 kDa (M Dialysis AB, Stockholm, Sweden). It was highly encouraged, but not mandatory, to insert these at the same time as the EVD, when ICP monitoring was considered. The MD catheter was placed via a burr-hole, adjacent to the EVD in normal-appearing brain tissue in the right frontal lobe. The MD was perfused by means of a microinjection pump (106 MD Pump, M Dialysis AB) at a rate of 0.3 µL/min with sterile artificial cerebrospinal fluid containing — NaCl 147 mmol/L (mM), KCl 2.7 mM, CaCl_2_ 1.2 mM, and MgCl_2_ 0.85 mM. Cerebral interstitial glucose, pyruvate, lactate, and urea were estimated hourly, using a CMA 600 analyzer or the ISCUSflex Microdialysis Analyzer (M Dialysis AB). The MD urea was monitored to validate catheter performance [20]. Total imprecision coefficient of variation was < 10% for all analytes.

Mean daily values for ICP, CPP, CPPopt, MD-glucose, MD-pyruvate, MD-lactate, and MD-lactate-pyruvate-ratio (LPR) were evaluated the first 10 days post-ictus for those with favorable and unfavorable outcome in the Odin software. The burden of ICP- and CPP-insults were similarly calculated during the first 10 days for those with favorable and unfavorable outcome. The burden of ICP-insults with fixed targeted thresholds was calculated as the percentage of monitoring time above (i) 20 mmHg and (ii) 25 mmHg. The threshold at 20 mmHg was chosen in accordance with our management protocol and 25 mmHg was chosen as a “severe insult.” The burden of CPP-insults was calculated as the percentage of monitoring time below (i) 60 mmHg, (ii) 70 mmHg, (iii) 80 mmHg, and (iv) 90 mmHg. The threshold at 60 mmHg was chosen in accordance with our management protocol and the threshold was then increased stepwise with 10 mmHg in accordance with a previous study [26]. The burden of autoregulatory CPPopt-insults was calculated as the difference between actual CPP and calculated CPPopt (∆CPPopt), and categorized as (i) hypoperfusion, ∆CPPopt <  − 10 mmHg and (ii) hyperperfusion, ∆CPPopt > 10 mmHg and lastly (iii) optimal, ∆CPPopt within ± 10 mmHg.

Furthermore, the energy metabolic pattern was classified as “poor cerebral substrate supply” (MD-LPR > 40 and concurrent MD-pyruvate < 120 µM) and “cerebral mitochondrial dysfunction” (MD-LPR > 40 and concurrent MD-pyruvate > 120 µM). The percent of these cerebral states was evaluated the first 10 days post-ictus for those with favorable and unfavorable outcome. The MD-LPR threshold of 40 for metabolic disturbances was chosen in accordance with the consensus statement 2014 [13]. The MD-pyruvate threshold of 120 µM was chosen because this is the highest pyruvate value for ischemic and the lowest value for non-ischemic cerebral conditions according to previous studies [19, 23].

The 10-day period was divided into three phases — (i) early phase (day 1 to 3), (ii) early vasospasm phase (day 4 to 6.5), and (iii) late vasospasm phase (day 6.5 to 10). The vasospasm phase (day 4 to 10) was hence split into two equally long periods of time. Mean values and the percent of monitoring time above/below the thresholds mentioned above were calculated for each phase in the Odin software.

### Statistical analysis

The analysis aimed primarily to determine the association of insults of ICP- and CPP-thresholds with cerebral energy metabolism and secondarily the association of these insults with clinical outcome.

Nominal, ordinal, and continuous variables were described as numbers or proportions, medians with interquartile range (IQR), and mean values with standard deviation (SD).

The association among ICP- (% above 20 mmHg and 25 mmHg), fixed CPP- (% below 60 mmHg, 70 mmHg, 80 mmHg, and 90 mmHg), and autoregulatory CPPopt-thresholds (∆CPPopt <  − 10 mmHg, ∆CPPopt > 10 mmHg, and ∆CPPopt within the optimal ± 10 mmHg) with cerebral energy metabolism was evaluated with univariate analysis (Spearman) for all three phases. Multiple linear regression analyses were performed with MD-LPR as the dependent variable in all phases. Age and GCS M at admission were included as baseline explanatory variables together with the ICP- and CPP-insult that had the strongest association with MD-LPR in the univariate analyses. Log_10_ transformation of MD-LPR was done to optimize the regressions due to the skewness of the data.

The association between the ICP-, CPP-, and CPPopt-insults and the MD-variables with clinical outcome was evaluated with the Spearman’s rank correlation test. No multiple logistic regression analyses for clinical outcome were done due to the limited number of patients with favorable outcome (*n* = 16).

A *p*-value < 0.05 was considered statistically significant. The statistical analyses were performed in SPSS version 28 (IBM Corp, Armonk, NY, USA).

## Results

### Patients, admission variables, treatments, and clinical outcome

Seventy-five patients were included. The majority were female (75%), mean age was around 60 years old, and typically had severe injuries based on the World Federation of Neurosurgical Societies (WFNS) and Fisher grade (Table 1). The aneurysm was more often located in the anterior (81%) than the posterior (19%) circulation and most patients were treated with endovascular embolization (79%) rather than clipping (19%). One in four patients developed DIND. Fifteen percent of the patients were treated with thiopental and 11% with decompressive craniectomy. Thiopental was initiated on day 5 in mean (range 2–11) due to ICP-problems with smaller infarctions in 8 cases and generalized edema in 3 cases. DC surgery was performed on day 5 in mean (range 2–10) due to ICP-problems with media infarction in 6 cases and generalized edema in 2 cases. After 1 year, 24% of the patients were deceased and 22% had recovered favorably and 78% unfavorably.

**Table 1:** Demography, admission status, treatments, and clinical outcome

Patients, *n* (%)	75 (100)
Age, mean (± SD)	59 ± 11
Male/female, *n* (%)	19/56 (25/75)
GCS M at admission, median (IQR)	5 (5–6)
WFNS grade IV-V/I-III, *n* (%)	59/15 (79/21)
Fisher grade, median (IQR)	4 (3–4)
Aneurysm location, anterior/posterior, *n* (%)	61/14 (81/19)
Embolization/clip ligation/no treatment, *n* (%)	59/14/2 (79/19/3)
DIND, *n* (%)	20 (27)
Thiopental, *n* (%)	11 (15)
DC, *n* (%)	8 (11)
GOS-E, median (IQR)	2 (3–4)
Mortality, *n* (%)	17 (24)
Favorable outcome, *n* (%)	16 (22)

*DC* decompressive craniectomy, *DIND* delayed ischemic neurological deficit, *GCS M* Glasgow Coma Scale Motor score, *GOS-E* Glasgow Outcome Scale-Extended, *IQR* interquartile range, *WFNS* World Federation of Neurosurgical Societies

### Cerebral physiological variables and insults the first 10 days after ictus

**Fig. 1:**
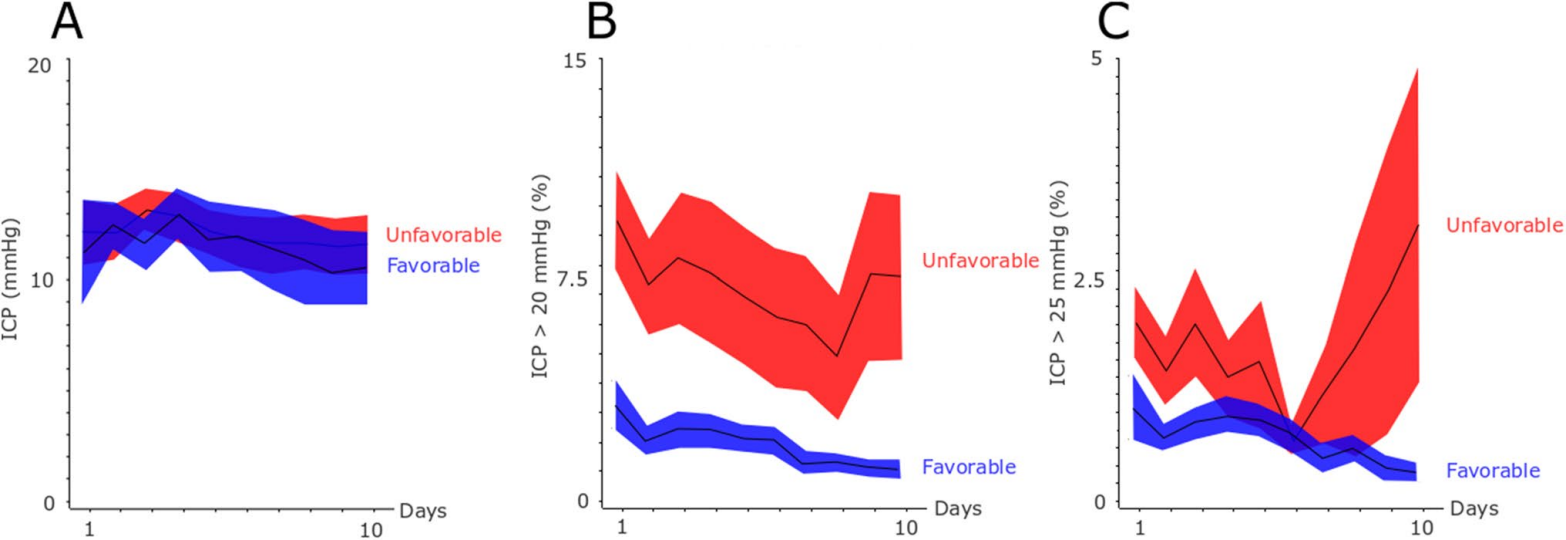
**A**–**C** Mean ICP and ICP-insults with fixed thresholds above 20 and 25 mmHg in relation to clinical outcome the first 10 days after ictus. The figure demonstrates mean daily values (95% *CI*) of ICP **A** and the percentage of ICP above 20 mmHg **B** and 25 mmHg **C** for those with favorable and unfavorable outcome the first 10 days after ictus. *CI* confidence interval, *ICP* intracranial pressure

**Fig. 2:**
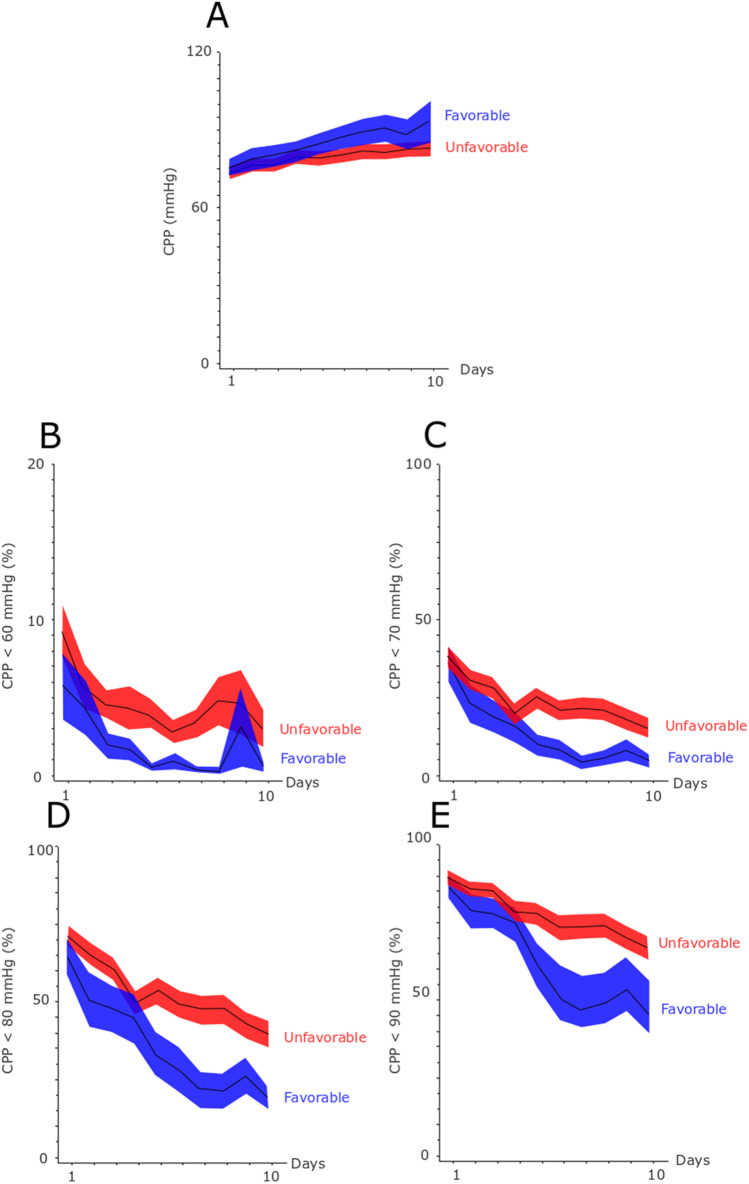
**A**–**E** Mean CPP and the burden of CPP-insults to different fixed thresholds in relation to clinical outcome the first 10 days after ictus. The figure demonstrates mean daily values (95% *CI*) of CPP **A** and the percentage of CPP below 60 mmHg **B**, 70 mmHg **C**, 80 mmHg **D**, and 90 mmHg **E** for those with favorable and unfavorable outcome the first 10 days after ictus. *CI* confidence interval, *CPP* cerebral perfusion pressure

**Fig. 3:**
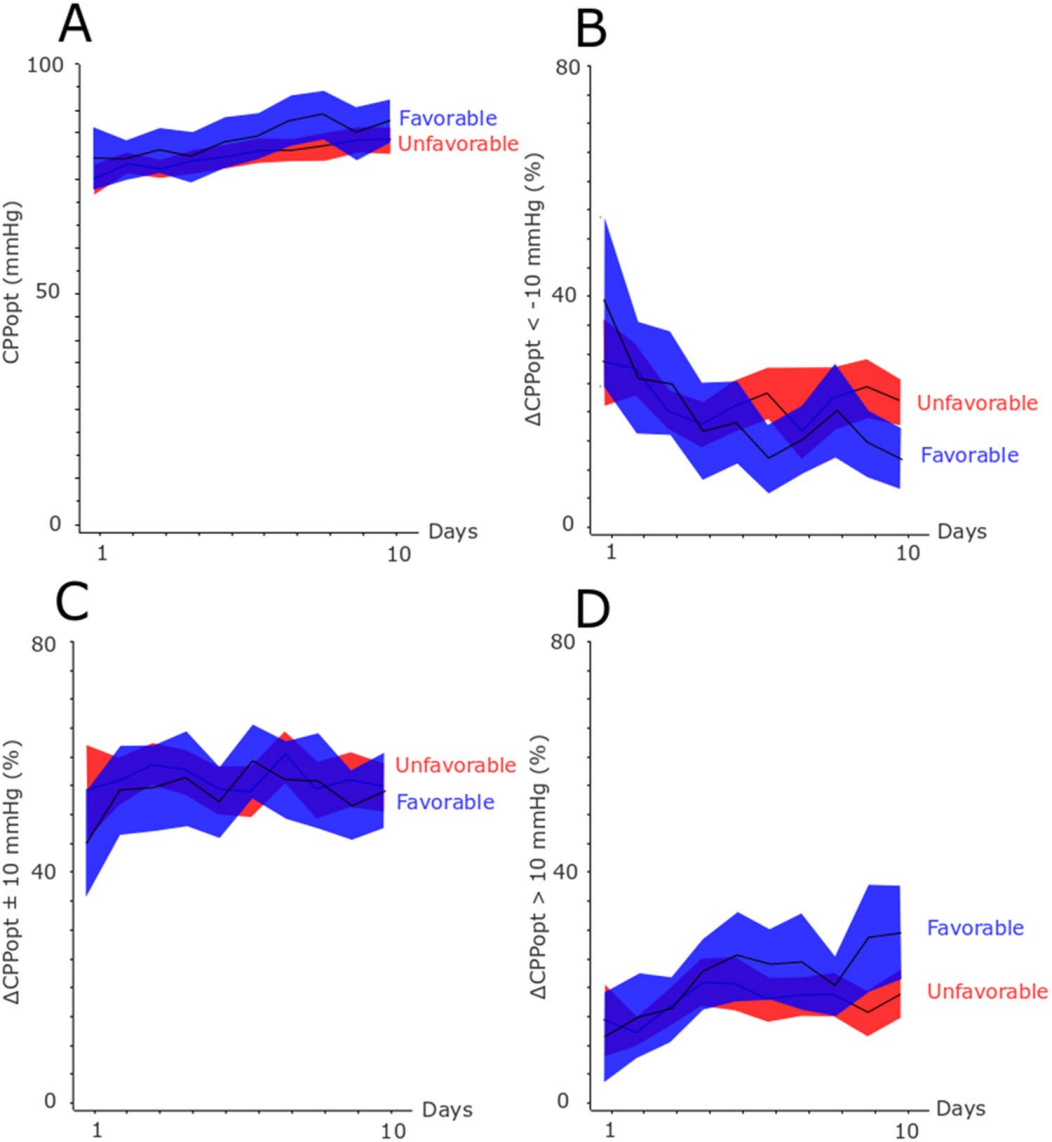
**A**–**D** Mean CPPopt and the burden of ∆CPPopt-insults in relation to clinical outcome the first 10 days after ictus. The figure demonstrates mean daily values (95% *CI*) of CPPopt **A** and the percentage of ∆CPP <  − 10 mmHg **B**, ∆CPP ± 10 mmHg **C**, and ∆CPP > 10 mmHg **D** for those with favorable and unfavorable outcome the first 10 days after ictus. *CI* confidence interval; *CPP* cerebral perfusion pressure, *CPPopt* optimal cerebral perfusion pressure, *∆CPP* CPP-CPPopt

**Fig. 4:**
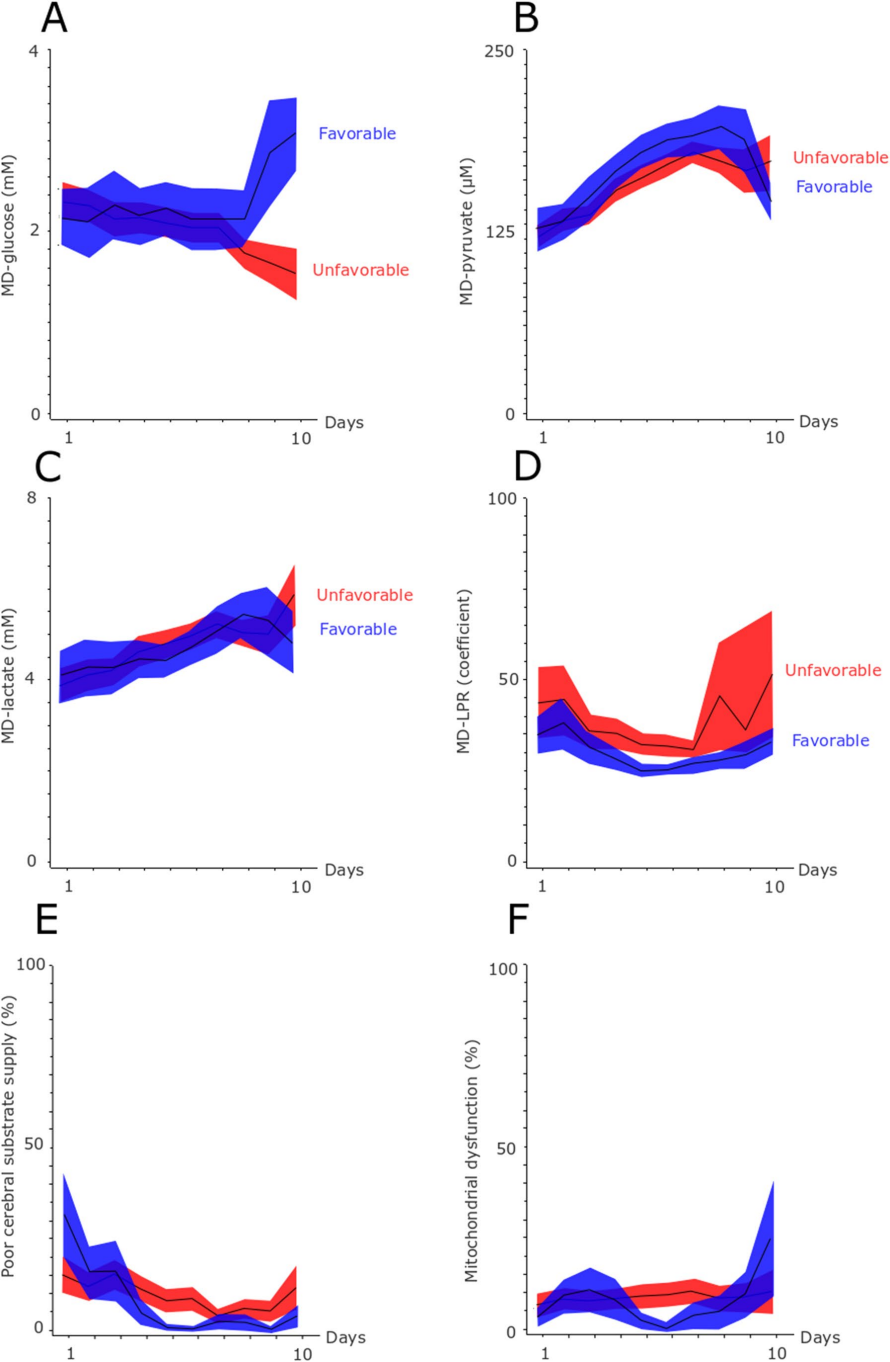
Cerebral energy metabolism in relation to clinical outcome the first 10 days after ictus. The figure demonstrates mean daily values (95% *CI*) of MD-glucose **A**, MD-pyruvate **B**, MD-lactate **C**, MD-LPR **D**, and the burden (%) of poor cerebral substrate supply **E** and mitochondrial dysfunction **F** for those with favorable and unfavorable outcome the first 10 days after ictus. Poor cerebral substrate supply was defined as MD-LPR > 40 and concurrent MD-pyruvate < 120 µM, whereas cerebral mitochondrial dysfunction was defined as MD-LPR > 40 and concurrent MD-pyruvate > 120 µM. The MD-LPR threshold at 40 for metabolic disturbances was chosen in accordance with the consensus statement 2014 [13]. The MD-pyruvate threshold at 120 µM was chosen as this is the highest pyruvate value for ischemic and the lowest value for non-ischemic cerebral conditions according to previous studies [19, 23]. *CI* confidence interval, *LPR* lactate-/pyruvate-ratio, *MD* microdialysis

The physiological variables and the insult burden are described in Figs. 1, 2, 3,  and 4. Mean ICP was around 10 mmHg and the percentage of ICP above 20 and 25 mmHg was approximately 5 and 2.5%, respectively (Fig. 1A–C). Mean CPP was around 70 mmHg in the early course and gradually increased to 90 mmHg in the late vasospasm phase (Fig. 2A–E). Correspondingly, the percent of CPP below 60 mmHg, 70 mmHg, 80 mmHg, and 90 mmHg gradually decreased the first 10 days. CPPopt remained at approximately 80–90 mmHg over the first 10 days (Fig. 3A–D). The percent of ∆CPPopt <  − 10 mmHg gradually decreased, whereas the percent of ∆CPPopt > 10 mmHg increased during the first 10 days.

Mean MD-glucose was around 2 mM and increased for those with favorable outcome, but decreased for those with unfavorable outcome in the late vasospasm phase (Fig. 4A–F). MD-pyruvate was around 125 μM at admission and increased to slightly above 200 μM in the vasospasm phase. Mean MD-lactate was 4 mM at admission and increased to 5 mM in the late vasospasm phase. MD-LPR was mostly between 25 and 50. The percent of MD pattern indicative of poor cerebral substrate supply was around 25% in the early course and gradually decreased to 5–10%. The pattern indicative of mitochondrial dysfunction increased slightly from 5% in the early phase to around 10% in the late vasospasm phase.

### Insults to ICP-, CPP-, and CPPopt-thresholds in relation to cerebral energy metabolism

For fixed ICP-thresholds, in the early phase (Table 2), higher percent of ICP > 20 mmHg correlated with a lower MD-pyruvate (*r* =  − 0.27, *p* < 0.05). In the early vasospasm phase, higher percent of ICP > 20 mmHg was associated with lower MD-glucose (*r* =  − 0.27, *p* < 0.05) and higher MD-LPR (*r* = 0.23, *p* < 0.05). Higher percent of ICP > 25 mmHg also correlated with a lower MD-glucose (*r* =  − 0.23, *p* < 0.05. In the late vasospasm phase, higher percent of ICP > 20 mmHg was associated with lower MD-glucose (*r* =  − 0.27, *p* < 0.05), higher MD-LPR (*r* = 0.25, *p* < 0.05), increased burden of poor cerebral substrate supply (*r* = 0.37, *p* < 0.001), and increased burden of mitochondrial dysfunction (*r* = 0.24, *p* < 0.05). In the same phase, higher percent of ICP > 25 mmHg was associated with increased MD-LPR (*r* = 0.26, *p* < 0.05), increased burden of poor cerebral substrate supply (*r* = 0.33, *p* < 0.01), and increased burden of mitochondrial dysfunction (*r* = 0.24, *p* < 0.05).

**Table 2:** Fixed intracranial pressure targets in relation to cerebral energy metabolism and clinical outcome after aneurysmal subarachnoid hemorrhage — a Spearman’s rank correlation analysis

	Early phase		Early vasospasm phase		Late vasospasm phase	
	ICP > 20 mmHg	ICP > 25 mmHg	ICP > 20 mmHg	ICP > 25 mmHg	ICP > 20 mmHg	ICP > 25 mmHg
MD-glucose	− 0.12	− 0.10	− ***0.27***^***a***^	− ***0.23***^***a***^	− ***0.27***^***a***^	− 0.16
MD-pyruvate	− ***0.27***^***a***^	− 0.21	− 0.10	− 0.11	− 0.18	− 0.17
MD-lactate	− 0.17	− 0.05	0.02	0.00	0.09	0.10
MD-LPR	0.05	0.06	***0.23***^***a***^	0.20	***0.25***^***a***^	***0.26***^***a***^
Poor cerebral substrate supply	− 0.00	− 0.01	0.06	0.02	***0.37***^***c***^	***0.33***^***b***^
Mitochondrial dysfunction	− 0.08	− 0.07	0.14	0.16	***0.24***^***a***^	***0.24***^***a***^
GOS-E	− 0.19	− 0.09	− 0.02	− 0.14	− 0.06	− 0.04

^a^*p*-value < 0.05, ^b^*p*-value < 0.01, ^c^*p*-value < 0.001. Bold and italics indicate statistical significance. *GOS-E* Glasgow Outcome Scale-Extended, *ICP* intracranial pressure, *LPR* lactate-pyruvate-ratio, *MD* microdialysis

For fixed CPP-thresholds (Table 3), higher percent of CPP below 90 mmHg correlated with lower MD-glucose (*r* =  − 0.24, *p* < 0.05) in the early phase and a higher percent of CPP below 60 mmHg, 70 mmHg, 80 mmHg, and 90 mmHg all correlated with increased burden of poor cerebral substrate supply (*r* ≈ 0.3 and *p* < 0.05 in all four correlations) in the late vasospasm phase.

**Table 3:** Fixed cerebral perfusion targets in relation to cerebral energy metabolism and clinical outcome after aneurysmal subarachnoid hemorrhage — a Spearman’s rank correlation analysis

	Early phase				Early vasospasm phase				Late vasospasm phase			
	CPP < 60 mmHg	CPP < 70 mmHg	CPP < 80 mmHg	CPP < 90 mmHg	CPP < 60 mmHg	CPP < 70 mmHg	CPP < 80 mmHg	CPP < 90 mmHg	CPP < 60 mmHg	CPP < 70 mmHg	CPP < 80 mmHg	CPP < 90 mmHg
MD-glucose	− 0.04	− 0.13	− 0.20	− ***0.24***^***a***^	− 0.11	− 0.14	− 0.11	− 0.08	− 0.15	− 0.08	− 0.04	− 0.05
MD-pyruvate	− 0.03	− 0.11	− 0.03	− 0.11	− 0.05	− 0.07	− 0.07	− 0.07	− 0.05	− 0.01	− 0.01	0.03
MD-lactate	− 0.06	− 0.06	− 0.02	0.02	− 0.03	0.00	0.04	0.05	0.12	0.10	0.09	0.11
MD-LPR	− 0.05	− 0.01	0.04	0.09	0.09	0.11	0.13	0.12	0.11	0.07	0.05	0.03
Poor cerebral substrate supply	− 0.03	− 0.03	0.01	0.08	0.06	0.10	0.10	0.13	***0.29***^***a***^	***0.30***^***b***^	***0.29***^***a***^	***0.28***^***a***^
Mitochondrial dysfunction	− 0.04	− 0.01	− 0.00	0.10	0.05	0.11	0.15	0.15	0.03	0.07	0.07	0.06
GOS-E	− 0.02	− 0.02	− 0.02	− 0.01	− ***0.34***^***b***^	− ***0.30***^***a***^	− ***0.27***^***a***^	− ***0.28***^***a***^	− ***0.33***^***b***^	− ***0.35***^***b***^	− ***0.36***^***b***^	− ***0.38***^***c***^

^a^*p*-value < 0.05, ^b^*p*-value < 0.01, ^c^*p*-value < 0.001. Bold and italics indicate statistical significance. *CPP* cerebral perfusion pressure, *GOS-E* Glasgow Outcome Scale-Extended, *LPR* lactate-pyruvate-ratio, *MD* microdialysis

For the autoregulatory CPP-thresholds (Table 4), a higher percent of ∆CPPopt ± 10 mmHg correlated with higher MD-LPR (*r* = 0.26, *p* < 0.05) and higher burden of poor cerebral substrate supply (*r* = 0.31, *p* < 0.01) in the early phase. Higher percent of ∆CPPopt > 10 mmHg was also associated with a lower burden of poor cerebral substrate supply (*r* =  − 0.28, *p* < 0.05) and lower burden of mitochondrial dysfunction (*r* =  − 0.25, *p* < 0.05) in the late vasospasm phase, but there was otherwise no association with cerebral energy metabolism.

**Table 4:** Autoregulatory cerebral perfusion targets in relation to cerebral energy metabolism and clinical outcome after aneurysmal subarachnoid hemorrhage — a Spearman’s rank correlation analysis

	Early phase			Early vasospasm phase			Late vasospasm phase		
	∆CPPopt < − 10 mmHg	∆CPPopt ± 10 mmHg	∆CPPopt > 10 mmHg	∆CPPopt < − 10 mmHg	∆CPPopt ± 10 mmHg	∆CPPopt > 10 mmHg	∆CPPopt < − 10 mmHg	∆CPPopt ± 10 mmHg	∆CPPopt > 10 mmHg
MD-glucose	− 0.02	− 0.19	0.15	− 0.02	− 0.03	0.05	− 0.04	0.12	− 0.11
MD-pyruvate	0.10	− 0.19	0.09	− 0.13	− 0.07	0.22	− 0.13	0.08	− 0.01
MD-lactate	− 0.01	0.15	− 0.07	0.01	− 0.09	0.12	0.02	0.01	− 0.16
MD-LPR	− 0.05	***0.26***^***a***^	− 0.11	0.15	− 0.06	− 0.04	0.13	− 0.09	− 0.14
Poor cerebral substrate supply	− 0.17	***0.31***^***b***^	− 0.08	0.15	− 0.03	− 0.18	0.21	0.07	− ***0.28***^***a***^
Mitochondrial dysfunction	− 0.11	0.15	− 0.06	0.11	− 0.13	− 0.09	0.11	− 0.11	− ***0.25***^***a***^
GOS-E	0.00	− 0.08	− 0.01	− ***0.35***^***b***^	0.13	***0.31***^***b***^	− ***0.34***^***b***^	0.11	***0.31***^***b***^

^a^*p*-value < 0.05, ^b^*p*-value < 0.01, ^c^*p*-value < 0.001. Bold and italics indicate statistical significance. *CPP* cerebral perfusion pressure, *CPPopt* optimal cerebral perfusion pressure, *∆CPPopt* CPP-CPPopt, *GOS-E* Glasgow Outcome Scale-Extended, *LPR* lactate-pyruvate-ratio, *MD* microdialysis

In a multiple linear regression analyses (Table 5), a higher percent of CPP < 60 mmHg (*β* = 0.24, *p* < 0.05) and younger age (*β* =  − 0.25, *p* < 0.05) were independently associated with a higher MD-LPR (log_10_ transformed) in the late vasospasm phase, whereas ICP > 25 mmHg and GCS M at admission were not associatied with MD-LPR. In another regression, in which the autoregulatory thresholds ∆CPPopt > 10 mmHg replaced the fixed CPP-threshold CPP < 60 mmHg, deviation from the autoregulatory CPP-threshold was not associated with MD-LPR. Similar multiple linear regressions for MD-LPR in the early phase and the early vasospasm phase were not significant (data not shown).

**Table 5:** Explanatory variables for lactate-pyruvate ratio in the late vasospasm phase in aneurysmal subarachnoid hemorrhage — a multiple linear regression analysis

Variables	*β* (95% *CI*)	*p*
Fixed CPP-thresholds		
Age	− 0.25 (( −)1.25–0.00)	***0.03***
GCS M at admission	− 0.05 (( −)0.29–0.20)	0.70
CPP < 60 mmHg	0.24 (0.00–0.49)	***0.05***
ICP > 25 mmHg	0.07 (( −)0.13–0.26)	0.60
Autoregulatory CPP-thresholds		
Age	− 0.22 (( −)0.45–0.00)	0.06
GCS M at admission	− 0.10 (( −)0.35–0.15)	0.42
∆CPPopt > 10 mmHg	− 0.19 (( −)0.47–0.00)	0.10
ICP > 25 mmHg	0.14 (( −)0.09–0.43)	0.25

Regression 1, fixed CPP-thresholds. *R*^2^ = 0.18, ANOVA *p*-value = 0.009. Regression 2, autoregulatory CPP-thresholds. *R*^2^ = 0.16, ANOVA *p*-value = 0.016. Due to skewness of LPR, a log_10_ transformation was done for this variable. Bold and italics indicate statistical significance. *CI* confidence interval, *CPP* cerebral perfusion pressure, *CPPopt* optimal CPP, *GCS M* Glasgow Coma Scale Motor score, *ICP* intracranial pressure, ∆*CPPopt* CPP-CPPopt

### Insults to ICP-, CPP-, and CPPop-thresholds and MD-variables in relation to clinical outcome

Insults above the ICP-thresholds 20 mmHg and 25 mmHg were not associated with clinical outcome (Table 2). Higher percent of CPP below 60 mmHg, 70 mmHg, 80 mmHg, and 90 mmHg was associated with unfavorable outcome in both the early vasospasm phase and the late vasospasm phase (Table 3). Higher burden of CPP above CPPopt correlated with better outcome in both vasospasm phases, higher burden of CPP below CPPopt had the opposite association, and CPP close to CPPopt was not associated with clinical outcome (Table 4).

For the MD-variables, a higher MD-pyruvate in the early vasospasm phase and lower MD-LPR, lower burden of poor cerebral substrate supply, and lower burden of mitochondrial dysfunction in the late vasospasm phase correlated with more favorable outcome (higher GOS-E) (Supplementary Table 1).

## Discussion

In the current study on 75 aSAH patients with ICP- and MD-monitoring, we found that ICP insults above 20 mmHg and 25 mmHg particularly in the vasospasm phases correlated with cerebral energy metabolic dysfunction, but not with clinical outcome. Furthermore, CPP below 60 mmHg in the late vasospasm phase was independently associated with energy metabolic dysfunction and also correlated with worse clinical outcome. CPP close to CPPopt was not associated with better cerebral energy metabolism and clinical outcome. Our findings support that avoiding intracranial hypertension above 20 mmHg and keeping CPP at least above 60 mmHg may benefit cerebral energy metabolism and clinical outcome in aSAH.

### ICP-threshold insults in relation to cerebral energy metabolism and clinical outcome

In univariate analyses, an increased burden of ICP-insults above 20 and 25 mmHg correlated with worse cerebral energy metabolism, characterized by a lower MD-glucose, higher MD-LPR, and higher burden of both poor cerebral substrate supply and mitochondrial dysfunction, most pronounced in both vasospasm phases. Insults above the 20 mmHg threshold rather than above 25 mmHg generally correlated more strongly with an increased burden of poor cerebral substrate supply, possibly due to the low frequency of insults above 25 mmHg. An explanation for the associations between ICP insults and cerebral energy metabolic dysfunction could be that high ICP lowers CPP, leading to cerebral ischemia and poor substrate delivery. This is consistent with the fact that ICP-insults were not independently associated with increased MD-LPR in the multiple regressions. However, we cannot exclude the possibility that ICP itself may influence cerebral metabolism independently of CPP, although the effect may have been too small to be significant in the multiple regression analysis. It has for example been shown that high ICP increases capillary transit time heterogeneity which leads to disturbances in the cerebral microcirculation with shunting of blood and suboptimal delivery of energy substrates [33]. The fact that high ICP also correlated with mitochondrial dysfunction may also reflect that poor cerebral energy metabolism generates cerebral swelling and, per se, contributes to intracranial hypertension.

In the current study, there was no association between the burden of ICP insults above 20 or 25 mmHg and clinical outcome. A potential explanation is that high ICP was actively treated and that we only included patients with MD monitoring the first 10 days, resulting in a small, homogeneous patient group that was for the most part severely injured. In previous studies from our group with larger aSAH populations, higher percent of ICP-insults above 25 mm Hg was associated with clinical deterioration [22] and ICP-insults above 20 mmHg with unfavorable outcome [26]. However, there are also some other studies which have not shown an association between ICP-insults and unfavorable outcome [10, 32]. Furthermore, other approaches have been made to quantify the effects of intracranial hypertension. For example, the ICP dose, i.e., the combination of ICP intensity and the duration of that episode, has in one recent study been more strongly associated with unfavorable outcome than ICP above certain fixed thresholds [4]. On balance, despite the negative results in this paper, higher ICP for longer periods of time does seem to exert a negative effect on the brain and worsen outcome.

### CPP- and CPPopt-threshold insults in relation to cerebral energy metabolism and clinical outcome

In this study, CPP below 60 mmHg was indepently associated with a lower oxidative energy metabolism (higher LPR) and CPP below all four thresholds (60/70/80/90 mmHg) were associated with an increased burden of poor cerebral substrate supply in the late vasospasm phase. CPP below 90 mmHg was also slightly associated with a lower MD-glucose in the early phase. As a surrogate measure of CBF, CPP is flawed because it does not taking into account cerebrovascular resistance and reactivity. Intact cerebral autoregulation means that CBF remains constant over a wide range of CPP. Changes in CPP within these autoregulatory limits would not be expected to impact CBF and consequently cerebral energy metabolism. However, the cerebrovascular resistance rises in the vasospasm phase and the autoregulatory capacity is often disturbed after aSAH [17]. This increases the susceptibility for low CPP, leading to cerebral ischemia and poor cerebral substrate delivery, as was found in this study.

An earlier aSAH study on CBF by our group demonstrated that CPP values below CPPopt correlated with cerebral ischemia [15] and earlier TBI studies demonstrated that CPP deviations from CPPopt correlated with brain tissue hypoxia [14] and worse cerebral energy metabolism [30]. Unexpectedly, we did not find any association between ∆CPPopt-insults and worse cerebral energy metabolism. On the contrary, CPP within the suggested optimal interval (∆CPPopt ± 10 mmHg) correlated with higher MD-LPR and higher burden of poor cerebral substrate supply in the early phase in univariate analyses. In addition, ∆CPPopt > 10 mmHg correlated with lower burden of poor cerebral substrate supply and mitochondrial dysfunction in the late vasospasm phase, which rather indicates that high CPP was beneficial.

Furthermore, CPP deviation above CPPopt correlated with more favorable clinical outcome, CPP deviation below CPPopt had the opposite association, and CPP close to CPPopt had no association with clinical outcome. The interpretation of this is that CPPopt does not reflect “the optimal CPP-target” in aSAH, but rather that high CPP, per se, rather than close to CPPopt explained the association with better outcome. This is consistent with a previous larger study by our group, in which high CPP was beneficial but there was no association between the percent of CPP close to CPPopt and favorable outcome [26]. The negative findings for CPPopt in aSAH could be explained by that it is based on PRx, which is a global measure of pressure autoregulation, but vasospasm, DIND, energy metabolic disturbances, and infarctions may be focal events which may go undetected by PRx [7]. Furthermore, we have earlier hypothesized that PRx may drift towards zero in case of distal cerebral vasospasm and may then falsely underestimate disturbed autoregulation [15]. Finally, CPPopt might be relatively high in case of cerebral vasospasm. It is possible that CPP values in these patients were typically not high enough to fully explore the upper limit of the CPPopt curve, which led to an underestimation of the true CPPopt.

Regarding the fixed targeted thresholds and clinical outcome, an increased burden of CPP below 60 mmHg, 70 mmHg, 80 mmHg, and 90 mmHg in the late vasospasm phase correlated both with lower burden of poor cerebral substrate supply and with worse clinical outcome. This is consistent with previous larger studies by our group [22, 26]. This supports the notion that spontaneuous CPP elevation up to 100 mmHg should at least not be lowered. Our patients were only treated with inotropes or vasopressors to reach the lower CPP-threshold of 60 mmHg. Future studies are needed to determine if such agents should be used to target even higher CPP-values and if this would improve CBF, cerebral energy metabolism, and clinical outcome in aSAH.

Altogether, fixed rather than autoregulatory CPP-thresholds seemed superior and CPP-targets at least above 60 mmHg, particularly in the vasospasm phase when the cerebrovascular resistance is increased, seems beneficial for cerebral energy metabolism and clinical outcome.

### Limitations

First, the analysis of association between ICP- and CPP-insults in relation to clinical outcome needs to be interptreted with caution due to the limited number of patients and small proportion of cases with favorble outcome. Second, this was a single-center retrospective study based of patients with severe aSAH, which limits the external validity of our findings. Third, the validity of PRx and hence CPPopt has been questioned in case of an open EVD. However, we and others have found that ICP slow waves are preserved when the EVD is opened and PRx and CPPopt should then still be valid [2, 12]. Fourth, the MD measured cerebral energy metabolism in a focal area in normal-appearing brain tissue in the right frontal lobe in this study. In this focal area, several important pathomechanisms for energy metabolic disturbances may occur, related to, e.g., the initial injury insult, cortical spreading depolarization, oxygen diffusion limitations due to edema, focal vasospasm and autoregulatory disturbances, and mitochondrial dysfunction. Another MD location could have influenced the results, particularly between different vascular territories and in penumbral areas in proximity to an intracerebral hemorrhage [16, 29]. An earlier Xenon-CT-CBF and microdialysis study by our group showed that global cortical CBF correlated strongly with CBF around the microdialysis catheter placed in normal-appearing brain [21]. It has also previously been demonstrated that disturbances in MD-variables (lactate, LPR, and glycerol) were more strongly correlated with clinical outcome when measured in normal-appearing brain rather than in perilesional areas [16]. This may also indicate that the energy metabolic state in the normal-appearing brain better reflects the global state of the brain, which is also more relevant for long-term recovery. However, the focality of MD is still limiting, when data are used to predict such a complex measure as clinical outcome that is influenced both by global and multifocal injuries in addition to many other variables such as patient age, co-morbidities, and neurorehabilitation. The correlation analyses between the MD and clinical outcome should hence be analyzed cautiously. Fifth, due to an absence of U-shaped curve between CPP and PRx, CPPopt could only be calculated during 54% of the monitoring time the first 10 days, similar to earlier findings in aSAH [26] and TBI [30]. This limits the validity of the CPPopt analyses. Fifth, many statistical tests were performed, which increases the risk of type I error. However, it is also possible that that some tests did not reach statistical significance due to limited number of patients. The significant correlations were also weak or moderate, which warrants caution when interpreting the data. However, the univariate correlations were only expected to be weak/moderate, considering the vast amount of variables affecting, e.g., LPR.

## Conclusions

Keeping the intracranial pressure below 20 mmHg and the cerebral perfusion pressure above at least 60 mmHg, and possibly considerably higher during the vasospasm phase, may be beneficial for cerebral energy metabolism and clinical outcome. Cerebral perfusion pressure close to the autoregulatory-oriented targets was not associated with better cerebral energy metabolism and clinical outcome.

## Supplementary Information

Below is the link to the electronic supplementary material.

Supplementary file1 (DOCX 65 KB)

Supplementary file2 (DOCX 17 KB)

Supplementary file3 (DOCX 32 KB)
