# Supplementary material for: Intracranial pressure- and cerebral perfusion pressure threshold-insults in relation to cerebral energy metabolism in aneurysmal subarachnoid hemorrhage
Source: Acta Neurochir (Wien). 2022 Mar 1;164(4):1001–14. doi: 10.1007/s00701-022-05169-y (PMC8967735; doi:10.1007/s00701-022-05169-y)
Supplement: Supplementary file 1 — Supplementary file1 (DOCX 65 KB) [file 701_2022_5169_MOESM1_ESM.docx]

**Supplementary Figure 1. CONSORT diagram.**

aSAH = Aneurysmal subarachnoid hemorrhage. ICP = Intracranial pressure. MD = Microdialysis. SAH = Subarachnoid hemorrhage.
