# Supplementary material for: Intracranial pressure- and cerebral perfusion pressure threshold-insults in relation to cerebral energy metabolism in aneurysmal subarachnoid hemorrhage
Source: Acta Neurochir (Wien). 2022 Mar 1;164(4):1001–14. doi: 10.1007/s00701-022-05169-y (PMC8967735; doi:10.1007/s00701-022-05169-y)
Supplement: Supplementary file 2 — Supplementary file2 (DOCX 17 KB) [file 701_2022_5169_MOESM2_ESM.docx]

**Supplementary table 1. Cerebral microdialysis variables and clinical outcome**

| GOS-E | | | | | | |
| --- | --- | --- | --- | --- | --- | --- |
| Variables | Early phase | | Early vasospasm phase | | Late vasospasm phase | |
|  | r | p-value | r | p-value | r | p-value |
| MD-glucose | -0.09 | 0.47 | 0.03 | 0.80 | 0.04 | 0.74 |
| MD-pyruvate | 0.11 | 0.35 | 0.28 | ***0.02*** | 0.17 | 0.16 |
| MD-lactate | 0.10 | 0.40 | 0.04 | 0.76 | -0.14 | 0.26 |
| MD-LPR | 0.03 | 0.81 | -0.17 | 0.16 | -0.29 | ***0.01*** |
| Poor cerebral substrate supply | 0.09 | 0.46 | -0.18 | 0.12 | -0.28 | ***0.02*** |
| Mitochondrial dysfunction | 0.10 | 0.42 | -0.17 | 0.16 | -0.32 | ***0.006*** |

Poor cerebral substrate supply was defined as MD-LPR > 40 and concurrent MD-pyruvate < 120 µM, whereas cerebral mitochondrial dysfunction was defined as MD-LPR > 40 and concurrent MD-pyruvate > 120 µM. The MD-LPR threshold at 40 for metabolic disturbances was chosen in accordance with the consensus statement 2014 [13]. The MD-pyruvate threshold at 120 µM was chosen as this is the highest pyruvate value for ischemic and the lowest value for non-ischemic cerebral conditions according to previous studies [19,23].

CI = Confidence interval. GOS-E = Glasgow Outcome Scale-Extended. LPR = Lactate-/pyruvate-ratio. MD = Microdialysis.
